# Applying Cost-Sensitive Extreme Learning Machine and Dissimilarity Integration to Gene Expression Data Classification

**DOI:** 10.1155/2016/8056253

**Published:** 2016-08-23

**Authors:** Yanqiu Liu, Huijuan Lu, Ke Yan, Haixia Xia, Chunlin An

**Affiliations:** ^1^College of Information Engineering, China Jiliang University, Hangzhou 310018, China; ^2^College of Informatics, Zhejiang Sci-Tech University, Hangzhou 310014, China

## Abstract

Embedding cost-sensitive factors into the classifiers increases the classification stability and reduces the classification costs for classifying high-scale, redundant, and imbalanced datasets, such as the gene expression data. In this study, we extend our previous work, that is, Dissimilar ELM (D-ELM), by introducing misclassification costs into the classifier. We name the proposed algorithm as the cost-sensitive D-ELM (CS-D-ELM). Furthermore, we embed rejection cost into the CS-D-ELM to increase the classification stability of the proposed algorithm. Experimental results show that the rejection cost embedded CS-D-ELM algorithm effectively reduces the average and overall cost of the classification process, while the classification accuracy still remains competitive. The proposed method can be extended to classification problems of other redundant and imbalanced data.

## 1. Introduction

With the appearance of gene chips, the classification methodology for gene expression data is developed into molecule phase [1]. The classification of gene expression data represents a crucial component in next generation cancer diagnosis technology [2]. For a particular tumor tissue with a series of known features, scientists believe that the classification of the gene array tells important information for identifying the tumor type and consequently influences the treatment plan [3–5]. However, the gene expression data on the other hand is known as large-scale, highly redundant, and imbalanced data, usually with relatively small sample size. Specifically, the number of features can be a hundred times larger than the number of samples [6]. This particular property of the gene expression data makes most of the traditional classifiers, such as extreme learning machine (ELM) [7], support vector machine (SVM), and multilayer neural networks, face difficulty in producing accurate and stable classification result. In 2012, we presented the integrated algorithm of Dissimilar ELM (D-ELM) by selective elimination of ELM based on V-ELM, which provided stable classification results compared to individual ELMs [8, 9].

Besides the accuracy, classification cost is another important aspect in performance evaluation for classification problems. In the cancer diagnosis progress, the cost of classifying a patient with cancer into negative class (false-negative) is much higher than that of classifying a patient without cancer into positive class (false-positive) [10]. Both false-negative and false-positive cases are recognized as misclassification cases. However, the costs of false-negative can be human lives due to the wrong medical treatments. Besides the misclassification cost, in recent years, the rejection cost also catches people's attention for cost-sensitive classifier development [11]. By considering misclassification and rejection cost, the classifiers become more stable and reliable.

In this study, aiming at extending the D-ELM to increase its classification stability, we embedded misclassification costs into D-ELM and named the proposed extension as CS-D-ELM. Furthermore, we embed the rejection costs into the CS-D-ELM to increase the classification stability of the proposed algorithm. The rejection cost embedded CS-D-ELM algorithm achieves the minimum classification cost with competitive classification accuracy. We validated CS-D-ELM by several commonly used gene expression datasets and compared the experimental results of using D-ELM, CS-ELM, and CS-SVM. The results show that the CS-D-ELM and rejection cost embedded CS-D-ELM both effectively reduce the overall misclassification costs and consequently enhance the classification reliability.

The rest of the paper is organized as follows. Related works, such as ELM, extensions of ELM, and cost-sensitive classifiers, are introduced in Section 2. In Section 3, the proposed algorithm is described in detail. The original D-ELM algorithm is extended by embedding misclassification costs and rejection costs. The experimental results are shown in Section 4. Conclusion, limitation, and future works are stated in Section 5.

## 2. Related Work

### 2.1. Extreme Learning Machine (ELM)

In 2004, Huang et al. first proposed the extreme learning machine as a single-hidden layer feedforward neural network (SLFN) [12–14]. The most famous advantage of ELM is the one-step training process, which results in much faster learning speed compared with traditional machine learning techniques, such as multilayer neural networks or support vector machine (SVM). The SLFN can also be applied to other research fields [15]. However, problems arise while the classification accuracy performance of a single ELM is not stable. Integrated ELM algorithms are developed to solve the above problem. Wang et al. [16] proposed an upper integral network with extreme learning mechanism. The upper integral extracts the maximum potential of efficiency for a group of features with interaction. Lan et al. [17] presented an enhanced integration algorithm with more stable performance and higher classification accuracy for Ensemble of Online Sequential ELM (EOS-ELM). Tian et al. [18, 19] used the Bagging Integrated Model and the modified AdaBoost RT to modify the conventional ELM, respectively. Lu et al. [20] proposed several algorithms to reduce the computational cost of the Moore-Penrose inverse matrices for ELM. Zhang et al. [21] introduced an incremental ELM which combines the deep feature extracting ability of Deep Learning Networks with the feature mapping ability of the ELM. Cao et al. [22] presented the majority Voting ELM (V-ELM), and this algorithm is widely used in various fields. Lu et al. [8, 9] presented the integrated algorithms of Dissimilar ELM (D-ELM) which is more adaptive for different individual ELMs compared with [22].

### 2.2. Cost-Sensitive Classifiers

In most integrated algorithms, the possibilities of samples belonging to given classes are calculated before judging the class labels of samples. However, if there are two or more probabilities which are equal or close to each other, misclassification is likely to happen. Therefore, the misclassification cost issue is studied to improve the classification performance of integrated algorithms. Foggia et al. [23] proposed a method to calculate the misclassification value and false rejection value of multiexpert systems based on Bayesian decision rules. Experimental results showed that their method was optimal. In 2003, Zadrozny et al. [24] introduced cost-sensitive factors into machine learning techniques, which further reduced the classification costs. In 2010, Masnadi-Shirazi and Vasconcelos [25] introduced both the misclassification costs and rejection costs into SVM, which improved the performance of cost-sensitive SVM algorithms. In 2011, Fu [26] proposed a cost-sensitive classification algorithm named Cost-MCP Boost for multiclassification problems. The Cost-MCP Boost algorithm solved the cost merge problem while the multiclass cost-sensitive classifications were converted into two-class cost-sensitive classifications; and the classification results were determined by the classes with smaller misclassification costs. In 2013, Cao et al. [27] proposed an optimized cost-sensitive SVM to deal with the imbalanced data learning problem.

Embedding classification costs into the ELM improves both the classification accuracy and the stability of the classifier [28]. Lu et al. [29–31] proposed cost-sensitive ELM for gene expression data classification. Experimental results showed that the misclassification cost dropped drastically and the classification accuracy was raised. Zong et al. [32] and Mirza et al. [33] utilized a weighted ELM to deal with imbalanced data. By assigning different weights to samples following user instructions, the weighted ELM can be generalized to cost-sensitive ELM. Riccardi et al. [34] worked on a cost-sensitive AdaBoost algorithm which is based on ELM. The cost-sensitive ELM is used for ordinal regression, which turns out to produce competitive results. Most recently, Fu et al. [35] showed some experimental results on the stability and generalization of ELM. The study provides some useful guidelines to ensemble ELM with cost-sensitive factors to produce more stable classification results. Wang et al. [36] indicated that samples with higher fuzziness outputted by the classifier mean a bigger risk of misclassification. They proposed a fuzziness category based divide-and-conquer strategy to promote the classifier performance.

## 3. Cost-Sensitive Dissimilar Extreme Machine (CS-D-ELM)

The ultimate goal of this study is to minimize the conditional risk:

(1) $$\begin{array}{c} arg\min R\left(\;i\;|\;x\;\right)=arg\min\underset{j}{\sum }P\left(\;j\;|\;x\;\right)\cdot C\left(\;i,j\;\right), \end{array}$$

where *R*(*i*∣*x*) is the conditional probability risk that the sample* x* is classified into the class* I*. *P*(*j*∣*x*) is the probability that sample* x* belongs to the class* j*. *C*(*i*, *j*) is the risk that a sample belonging to class* j* is misclassified to class* i*, where* i* and* j* belong to the set {*C*_1_, *C*_2_,…, *C*_*m*_} and* m* is the class number.

### 3.1. The General Form of D-ELM

The D-ELM is an improved algorithm for majority Voting ELM [37]. It selects the most suitable ELM individuals after a training process in order to improve the consistency in the voting procedure and therefore increase the classification accuracy. For example, suppose there are* N* ELMs and* M* training samples available for initialization. We construct a dissimilarity matrix to eliminate inappropriate ELM individuals. The remaining ELMs are considered in consistent form and are able to provide more stable classification results. The dissimilarity matrix is defined by inconsistency degree of outputs. If the *i*th ELM and the* j*th ELM have equal judgement results of *f*_*ik*_ and *f*_*jk*_ for the sample* k*, respectively, we mark Dif(*f*_*ik*_, *f*_*jk*_) = 0, (*i* = 1,2,…, *N*; *j* = 1,2,…, *N*; *K* = 1,2,…, *M*). Otherwise, we mark Dif(*f*_*ik*_, *f*_*jk*_) = 1. Suppose that Div_*i*,*j*_ = ∑_*k*=1_^*N*^Dif(*f*_*ik*_, *f*_*jk*_) denotes the difference between the *i*th ELM and the* j*th ELM. The dissimilarity matrix is expressed as

(2) $$\begin{array}{c} Div=\left[\begin{array}{ccccc} Div_{1,1} & \cdots & Div_{1,j} & \cdots & Div_{1,N}\\ \vdots & & \vdots & & \vdots \\ Div_{i,1} & \cdots & Div_{i,j} & \cdots & Div_{i,N}\\ \vdots & & \vdots & & \vdots \\ Div_{N,1} & \cdots & Div_{N,j} & \cdots & Div_{N,N} \end{array}\right]. \end{array}$$

Obviously, Div is a matrix with zeros for all diagonal elements.

*η*_*i*_ denotes the dissimilarity between the *i*th ELM and the rest of the ELMs. It is defined as

(3) $$\begin{array}{c} \eta_{i}=\overset{N}{\underset{j=1}{\sum }}{Div}_{i,j}. \end{array}$$

The average classification accuracy is denoted as $\overset{-}{p}$. The ELM with smaller *η* value is eliminated under the condition of $0<\overset{-}{p}\leq 0.5$. And the ELM with bigger *η* value is eliminated under the condition of $0.5<\overset{-}{p}<1$. The remaining ELMs are selected to proceed to the voting procedure.

The overall D-ELM algorithm can be divided into three parts. First,* N* independent ELMs are trained using given data; and a number of ELMs are eliminated according to dissimilarity theory. Second, the remaining* K* ELMs are trained again using the same hidden layer node number and activation function. For each independent ELM input layer, hidden layer weights and bias are randomly generated and unrelated. Last, for each testing sample* tx*, the* K* independent ELMs can maximally predict* K* individual classification results. An initial empty vector (*W*_*k*,*tx*_(*c*_1_), *W*_*k*,*tx*_(*c*_2_),…, *W*_*k*,*tx*_(*c*_*m*_)) (*m* is the number of classes) is used to store the classification results of* tx* for the* K* ELMs. For example, for the* l*th (*l* ∈ [1,…, *K*]) ELM classifier, if the classification result of* tx* is *i* (*i* ∈ {*C*_1_, *C*_2_,…, *C*_*m*_}), then the following operations are carried out:

(4) $$\begin{array}{c} W_{K,tx}\left(\;i\;\right)=W_{K,tx}\left(\;i\;\right)+1. \end{array}$$

The final vector *W*_*k*,*tx*_ is obtained after all* K* ELMs are processed. We get the probability for each class in the classification result:

(5) $$\begin{array}{c} P\left(\;i\;|\;tx\;\right)=\frac{W_{K,tx}\left(i\right)}{K},\;i\in \left\{c_{1},c_{2},\ldots ,c_{m}\right\}. \end{array}$$

After calculating the conditional probability of the test sample* tx* by D-ELM, if* tx* is classified into* s*th class correctly, the probability that* tx* belongs to the* s*th class is bigger than the probability that it belongs to other classes; that is, there is an inequality:

(6) $$\begin{array}{c} P\left(\;s\;|\;tx\;\right)\geq \max{\left\{\;P\left(\;i\;|\;tx\;\right)\;\right\}}_{i\in \left[c_{1},c_{2},\ldots ,c_{m}\right]}. \end{array}$$

For example, in a two-class classification, the probabilities that a testing sample* tx* belongs to the positive class and negative class are *P*(*p*∣*tx*) = *W*_*k*,*tx*_(*p*)/*K* and *P*(*n*∣*tx*) = *W*_*k*,*tx*_(*n*)/*K*, respectively.

### 3.2. Embedding Misclassification Costs into D-ELM

For each test sample* tx*, it is not enough to only know the probability *P*(*j*∣*tx*) (*j* ∈ {*C*_1_, *C*_2_,…, *C*_*m*_}) of* tx*. When the cost is unequal, even if the inequality (6) is satisfied, we cannot determine the class label* s* of* tx*. Therefore, in this section, the asymmetric misclassification costs are embedded in order to improve D-ELM to CS-D-ELM.

Suppose the probability *P*(*j*∣*tx*) is calculated by the D-ELM method in Section 3.1; the class label of* tx* is determined by taking the least cost that* tx* belongs to a class* i*. The following equation is derived from (1):

(7) ty−arg mini⁡Ri ∣ tx=arg mini⁡∑jPj ∣ x·Ci,j.

All class labels of test samples are recalculated according to the principle of minimizing the average misclassification costs. Let $\bar{ty}$ be the real class label of sample* tx* after integrating the misclassification cost information of* tx*. After embedding the misclassification cost into the D-ELM, the classification results are as follows:

(8) ty¯ty¯1⋮ty¯N~=arg mini⁡Ri ∣ tx1⋮arg mini⁡Ri ∣ txN~=arg mini⁡∑jPj ∣ tx1·Ci,j⋮arg mini⁡∑jPj ∣ txN~·Ci,j,

where *P*(*j*∣*tx*) = *R*_*K*,*tx*_(*j*)/*K* (*j* ∈ {*C*_1_, *C*_2_,…, *C*_*m*_}) is the probability calculated by D-ELM in Section 3.1.

### 3.3. The CS-D-ELM Algorithm

A general form of CS-D-ELM algorithm can be described as follows:

(1) Set initial values for all* N* ELMs.
(2) Randomly generate the input layer parameters (*a*_*j*_^*i*^, *b*_*j*_^*i*^), *j* = 1,…, *L*  (*L* is the number of nodes in hidden layer), of the *i*th ELM.
(3) Calculate the hidden layer output matrix of the *i*th ELM.
(4) Calculate the *i*th output weights, that is, a target output matrix.
(5) Process the* N* ELMs using dissimilarity elimination, assuming that* k* ELMs are left after elimination.
(6) For the test samples, predict each class of* tx* by using the remaining* k* ELMs. Assuming* tx* belongs to class* j*, where *j* ∈ {*C*_1_, *C*_2_,…, *C*_*m*_}, we have *W*_*K*,*tx*_(*j*) = *W*_*K*,*tx*_(*j*) + 1.
(7) Calculate the probability that the test set belongs to each class *P*(*j*∣*tx*) = *R*_*K*,*tx*_(*j*)/*K*.
(8) Use (8) to calculate the true class labels.
(9) End.

### 3.4. Embedding Rejection Costs into CS-D-ELM

The samples with low classification reliability are more likely to be misclassified. In order to reduce the high cost of misclassification, rejection options are embedded into CS-D-ELM to prevent automatic classification of samples with low classification reliabilities. There are three kinds of rejection costs:

1. The costs that require further analysis for unclassified samples.
2. Loss of samples because of the rejection decision.
3. Both circumstances above.

The rejection cost is defined as follows. For a given small positive number *δ* (rejection threshold) and any test samples* tx* with the following equations being true:

(9) Rs ∣ tx<max⁡Ri ∣ txi∈c1,…,cm,i≠sftx=min⁡Ri ∣ txi∈c1,…,cm,i≠s−Rs ∣ tx,

if *f*(*tx*) ≥ *δ*, the test sample is classified into the* s*th class. If *f*(*tx*) < *δ*, the test sample is processed by rejection treatment.

For the two-class classification problems, embedded misclassification costs and rejection costs, given a test sample

(10) $$\begin{array}{c} tx=\left\{\;\left(\;tx_{1},ty_{1}\;\right),\ldots ,\left(\;tx_{i},ty_{i}\;\right),\ldots ,\left(\;tx_{\tilde{N}},ty_{\tilde{N}}\;\right)\;\right\}, \end{array}$$

where *tx*_*i*_ ∈ *R* ^*n*^ and  *ty*_*i*_ ∈ {*n*, *p*}, $i=1,\ldots ,\tilde{N}$; and the cost matrix is:

(11) $$\begin{array}{c} C=\left\{\;C\left(\;p,n\;\right),C\left(\;n,p\;\right),C\left(\;0,n\;\right),C\left(\;0,p\;\right)\;\right\}, \end{array}$$

where *C*(*p*, *n*) and *C*(*n*, *p*) are misclassification costs and *C*(0, *n*) and *C*(0, *p*) are rejection costs. *P*(0∣*x*) (rejection rate), *P*(*n*∣*x*), and *P*(*p*∣*x*) can be calculated on the basis of the rejection threshold *δ*. Then, the test sample is evaluated by calculating minimum average misclassified cost; that is,

(12) ty−arg mini⁡Ri ∣ tx=arg mini⁡∑jPi ∣ xi ∣ xCj,i,

where *i*, *j* ∈ {0, *n*, *p*}. The rejection threshold *δ* is determined on a case-by-case basis which depends on the specific dataset. An example of a particular *δ* value calculation can be found in Section 4.3.

## 4. Experiments and Analysis

### 4.1. Experimental Datasets

For the performance evaluation of the CS-D-ELM algorithm, we perform experiments on six reduced gene expression datasets, that is, Diabetes, Heart, Colon, Mushrooms, Protein, and Leukemia. All datasets are preprocessed by feature selection methods to reduce the data size. The Diabetes dataset includes 97 gene samples and each sample includes 8 gene expression features. The Heart dataset consists of 270 samples with 13 features. The Colon dataset consists of 62 samples with 52 features. The Protein dataset contains 334 samples with 73 gene features. The Mushroom dataset has 263 samples with 43 gene features. The Leukemia dataset includes 72 acute leukemia samples, and the expression data of each sample contains 7129 genes. The first five datasets belong to the two-class classification problem, while the Leukemia dataset belongs to the multiclass classification problem.  Table 1 concludes the information of all the six datasets.

### 4.2. Experimental Results of CS-D-ELM

The misclassification costs of false-positive and false-negative are set as *C*(1, − 1) = 1 and *C*(−1,1) = 5. The experiments are repeated 30 times in each dataset; and average result of the 30 times is recorded as the final experimental results. In every experiment, 300 samples are randomly selected as training set; and the remaining samples are used for testing purpose.

**Figure 1:**
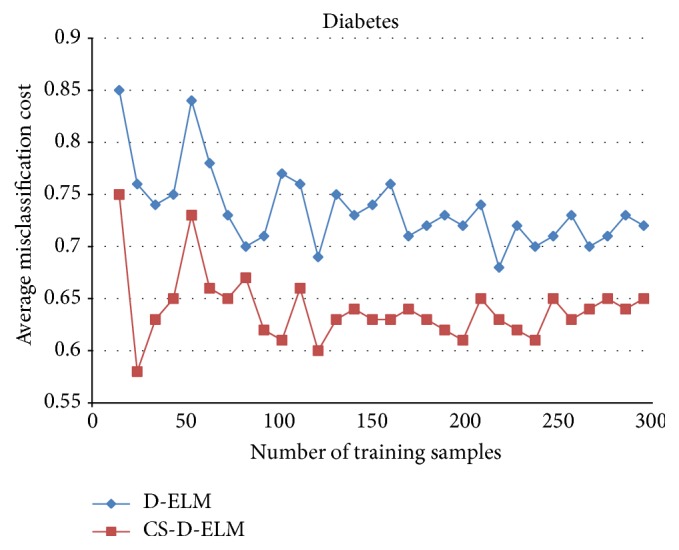
Average misclassification costs for Diabetes dataset.

**Figure 2:**
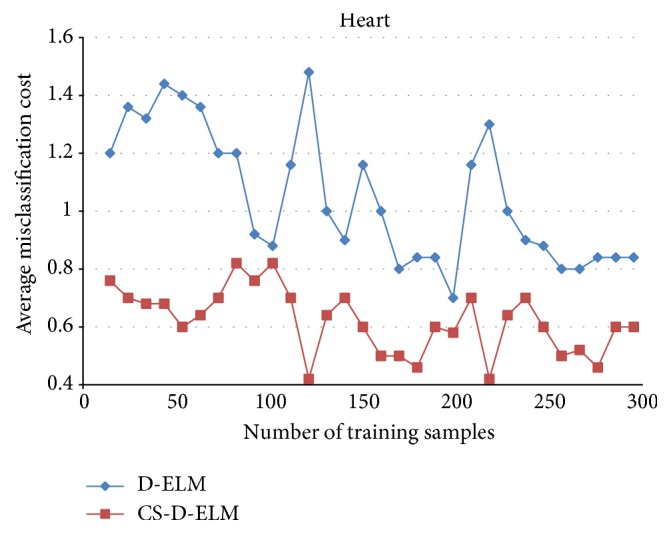
Average misclassification costs for Heart dataset.

**Figure 3:**
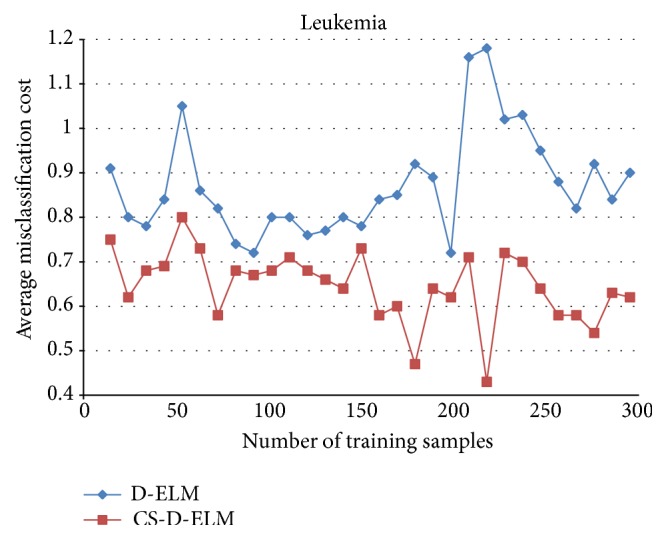
Average misclassification costs for Leukemia dataset.

Figures 1, 2, and 3 represent the average misclassification costs of D-ELM and CS-D-ELM on Diabetes, Heart, and Leukemia datasets, respectively. The average misclassification costs based on CS-D-ELM are lower than those based on D-ELM. While the number of training samples increases, the average misclassification cost decreases.

**Figure 4:**
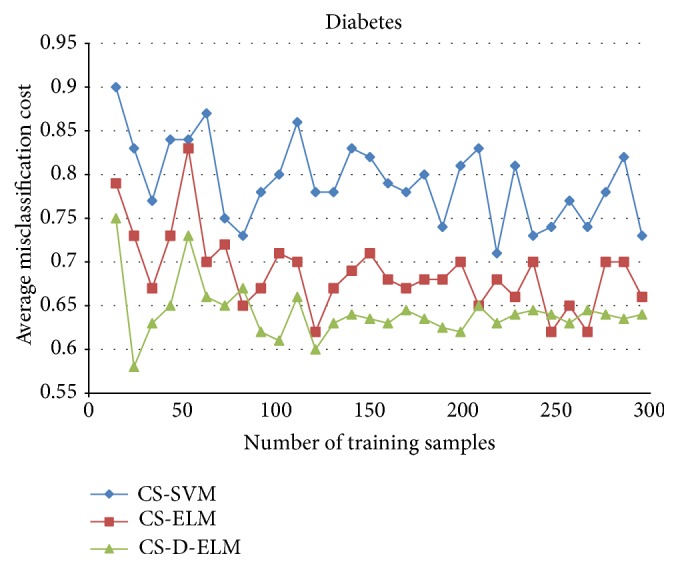
Comparison of average classification costs for Diabetes dataset.

**Figure 5:**
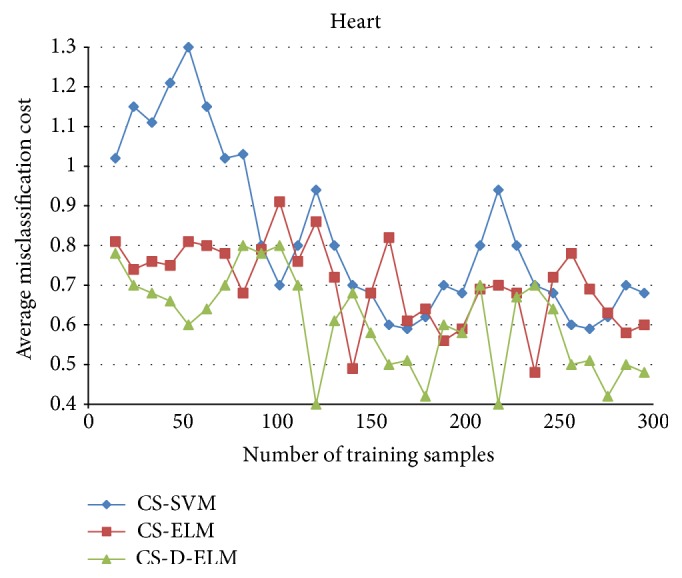
Comparison of average classification costs for Heart dataset.

**Figure 6:**
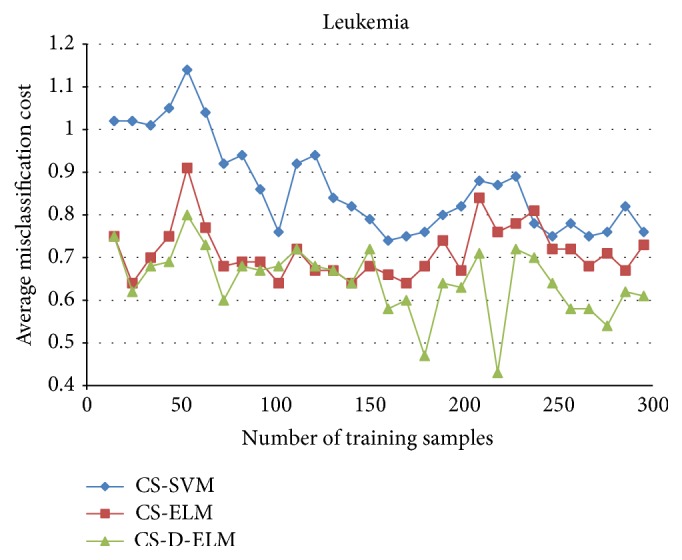
Comparison of average classification costs for Leukemia dataset.

To further verify the effectiveness of CS-D-ELM, we compared CS-D-ELM with cost-sensitive ELM (CS-ELM) and mature cost-sensitive support vector machine (CS-SVM) [27]. Figures 4, 5, and 6 represent the average misclassification costs of CS-ELM, CS-SVM, and CS-D-ELM on Diabetes, Heart, and Leukemia datasets, respectively. The average misclassification costs produced by CS-D-ELM are lower than those based on CS-ELM and CS-SVM.

### 4.3. Experimental Results of CS-D-ELM with Embedded Rejection Costs

Before embedding the rejection costs into the CS-D-ELM, an important preprocessing step must be performed, which is the rejection threshold determination. Taking the Heart dataset as an example, on the basis of the invariant cost matrix, we set the rejection costs to *C*(0,1) = *C*(1,0) = 0.2. Randomly selected 300 samples are treated as the training sample set; and the remaining samples are treated as testing sample set. Again, 30 independent experiments are performed with average misclassification costs recorded.

**Figure 7:**
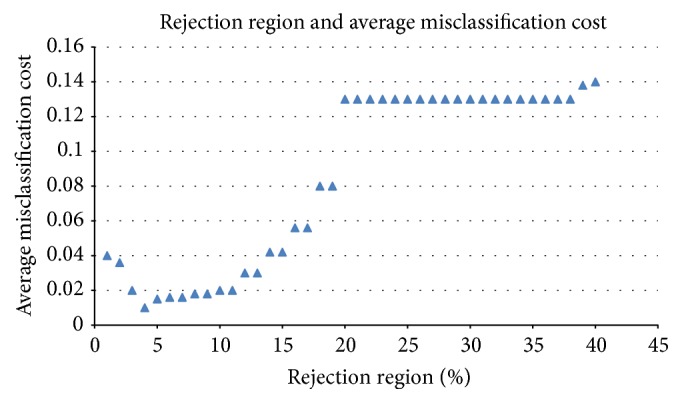
Relationship between the rejection threshold and the average misclassification costs.

**Figure 8:**
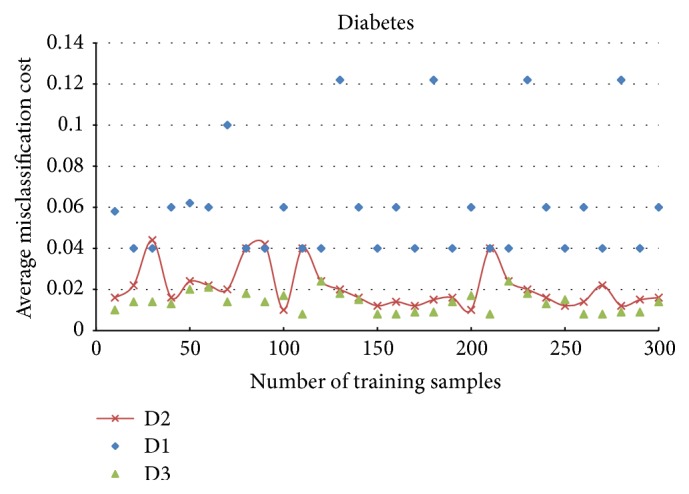
Comparison of embedding different cost-sensitive factors.

Figure 7 shows the relationship between the rejection threshold and the average misclassification costs. The average misclassification cost is minimized while rejection threshold *δ* equals 0.04. Using *δ* = 0.04 in the CS-D-ELM method, we get the results in Figure 8. In Figure 8, D1, D2, and D3 denote the results under the condition of not considering misclassification costs and embedding misclassification costs and rejection costs, respectively. The embedding of rejection costs reduces the average misclassification cost greatly. Considering that the D-ELM algorithm includes a voting process of ELMs to filter dissimilarity, the time performance of CS-D-ELM is 3-4 times slower than each individual ELM. A speed comparison between ELM, D-ELM, CS-D-ELM, and rejection cost embedded CS-D-ELM is shown in Table 2. We use a 32 Core@ 2.26 Hz Dell server machine with 128 G RAM. The voting process is conducted with 100 ELM individuals. Under the assumption that time is not the top priority in this study, the following conclusion can be drawn from this experiment: while the sample misclassification costs are not equal, the average misclassification can be effectively reduced by embedding rejection costs into CS-D-ELM.

In order to further verify the universal applicability of CS-D-ELM on gene expression data, we compare D-ELM, CS-D-ELM, and CS-D-ELM with embedded rejection costs on 4 datasets, namely, Leukemia, Colon, Mushroom, and Protein. The classification accuracies on the positive class and the negative class are denoted as NC and PC. In each dataset, the average value of 30 experiments is used as the experimental results. The NC values, PC values, G-means values, and the average misclassification costs when using the three algorithms are shown in Table 3.

As results shown in Tables 3 and 4, the G-means values are improved in CS-D-ELM and CS-D-ELM with embedded reject costs, and the effect of CS-D-ELM with embedded rejection costs is better. The average misclassification costs of CS-D-ELM are smaller than those of D-ELM in all datasets, indicating that CS-D-ELM can effectively reduce the misclassification costs in the classification process. The average misclassification costs of CS-D-ELM with embedded rejection costs are lower than those of the standard CS-D-ELM in all datasets. Again, the same conclusion can be drawn: when the misclassification costs of samples are not equal, rejection cost embedded CS-D-ELM can effectively reduce the average misclassification costs.

## 5. Conclusion and Discussion

The traditional classification algorithms are all based on the classification accuracy. When the misclassification costs are not equal, they cannot achieve the minimum average misclassification cost requirements in cost-sensitive classification process. This paper first reconstructs the classification results by introducing the probability estimation and misclassification costs into the classification process and proposes the cost-sensitive D-ELM algorithm which is called CS-D-ELM. Furthermore, by embedding the rejection costs, the average misclassification costs are further reduced.

For computational complexity evaluation, the time taken by the voting procedure is negligible as the training process of each ELM is the most costly part. The speed of the proposed algorithm depends on the number of parallelizable cores in the machine. In our case, a 32-core server machine is utilized to run the CS-D-ELM with 100 ELM individuals. The overall running time of the proposed algorithm is three to four times slower than each ELM individual. However, as the average running time for each ELM individual is generally less than one second, the overall running speed is still fast.

The embedding of misclassification costs and rejection costs is proved to be useful for classification cost reduction and accuracy improvement [8, 11, 30]. The way of embedding misclassification costs and rejection costs into the D-ELM can be employed for other cost-sensitive algorithms. As a future work, we are implementing a cost-sensitive rotational forest algorithm (CS-RoF) for gene expression data classification. Similar algorithms can also be extended for classification problems of other imbalanced datasets.

## Figures and Tables

**Table 1:** Datasets.

Datasets	Sample number	Feature number	Class distribution	
			Class name	Sample number
Diabetes	97	8	Relapse	46
			Nonrelapse	51
Heart	270	13	Negative	150
			Positive	120
Colon	62	52	Negative	19
			Positive	43
Mushroom	263	43	Negative	111
			Positive	152
Protein	334	73	Negative	215
			Positive	119
Leukemia	72	7129	ALL	24
			MLL	20
			AML	28

**Table 2:** Running time comparison between ELM, D-ELM, CS-D-ELM, and rejection cost embedded CS-D-ELM.

Dataset	Average running time for different algorithms (recorded in sec.)			
	ELM	D-ELM	CS-D-ELM	Rejection cost embedded CS-D-ELM
Diabetes	0.4312	1.4536	1.5330	1.5470
Heart	0.6670	1.8579	1.9353	1.9455
Colon	0.5183	1.6743	1.7458	1.7561
Mushroom	0.7214	1.8654	1.9583	1.9836
Protein	0.7551	2.0836	2.1349	2.2655
Leukemia	1.2319	3.9593	4.0346	4.1692

**Table 3:** NC value and PC value of D-ELM, CS-D-ELM, and embedded rejection costs into S-D-ELM.

Dataset	NC value			PC value		
	D-ELM	CS-D-LM	Rejection CS-D-LM	D-ELM	CS-D-LM	Rejection CS-D-LM
Leukemia	0.3815	0.4714	0.5874	0.9561	0.8626	0.8215
Colon	0.4132	0.5127	0.6322	0.9722	0.9152	0.8464
Mushroom	0.4325	0.5423	0.6929	1.0000	0.9605	0.9313
Protein	0.3237	0.4621	0.5433	0.9433	0.8956	0.7751

**Table 4:** Experiment results of G-means value and average misclassification costs.

Dataset	G-means value			Average misclassification costs		
	D-ELM	CS-D-LM	Rejection CS-D-LM	D-ELM	CS-D-LM	Rejection CS-D-LM
Leukemia	0.6416	0.6508	0.6714	0.4271	0.3522	0.2543
Colon	0.6852	0.7203	0.7313	0.3814	0.2232	0.2123
Mushroom	0.7125	0.7877	0.7922	0.1102	0.0755	0.0411
Protein	0.5333	0.7122	0.7328	0.4843	0.3812	0.2043
